# Deep Reinforcement Learning-Enabled Computation Offloading: A Novel Framework to Energy Optimization and Security-Aware in Vehicular Edge-Cloud Computing Networks

**DOI:** 10.3390/s25072039

**Published:** 2025-03-25

**Authors:** Waleed Almuseelem

**Affiliations:** Faculty of Computing and Information Technology (FCIT), University of Tabuk, Tabuk 47713, Saudi Arabia; waleedalmuseelem@ut.edu.sa

**Keywords:** autonomous vehicles, load balancing, task offloading, energy efficiency, vehicular edge-cloud computing, security, task caching, deep reinforcement learning

## Abstract

The Vehicular Edge-Cloud Computing (VECC) paradigm has gained traction as a promising solution to mitigate the computational constraints through offloading resource-intensive tasks to distributed edge and cloud networks. However, conventional computation offloading mechanisms frequently induce network congestion and service delays, stemming from uneven workload distribution across spatial Roadside Units (RSUs). Moreover, ensuring data security and optimizing energy usage within this framework remain significant challenges. To this end, this study introduces a deep reinforcement learning-enabled computation offloading framework for multi-tier VECC networks. First, a dynamic load-balancing algorithm is developed to optimize the balance among RSUs, incorporating real-time analysis of heterogeneous network parameters, including RSU computational load, channel capacity, and proximity-based latency. Additionally, to alleviate congestion in static RSU deployments, the framework proposes deploying UAVs in high-density zones, dynamically augmenting both storage and processing resources. Moreover, an Advanced Encryption Standard (AES)-based mechanism, secured with dynamic one-time encryption key generation, is implemented to fortify data confidentiality during transmissions. Further, a context-aware edge caching strategy is implemented to preemptively store processed tasks, reducing redundant computations and associated energy overheads. Subsequently, a mixed-integer optimization model is formulated that simultaneously minimizes energy consumption and guarantees latency constraint. Given the combinatorial complexity of large-scale vehicular networks, an equivalent reinforcement learning form is given. Then a deep learning-based algorithm is designed to learn close-optimal offloading solutions under dynamic conditions. Empirical evaluations demonstrate that the proposed framework significantly outperforms existing benchmark techniques in terms of energy savings. These results underscore the framework’s efficacy in advancing sustainable, secure, and scalable intelligent transportation systems.

## 1. Introduction

The rapid evolution of vehicular technologies, such as autonomous driving systems [1,2], a diverse range of vehicles, including electric cars, connected vehicles, and autonomous drones [3,4], has surged dramatically, leading to the emergence of numerous computationally intensive intelligent applications [5,6]. These applications include real-time traffic analytics, high-definition mapping, vehicle-to-everything communication, autonomous navigation, and advanced driver assistance systems [7]. Such applications impose stringent latency and computational demands, yet vehicles often face resource constraints, including limited onboard processing power and energy efficiency challenges [8]. Consequently, managing these computationally expensive and latency-sensitive tasks becomes a significant hurdle. The computational offloading concept has emerged to address this gap by migrating resource-intensive tasks to a more powerful remote server; therefore, vehicles can achieve better performance and efficiency [9,10].

Vehicular Cloud Computing (VCC) was initially proposed to enhance application performance and reduce power consumption by providing vehicles with access to versatile computing, storage, and service capabilities [11]. However, Centralized VCC introduces critical issues such as high communication latency, network instability, security, and bandwidth limitations, which contradict the fundamental requirements of emerging vehicular applications, thereby hindering their advancement [12]. Another practical solution emerges, named the vehicular edge computing (VEC) paradigm, where complex tasks will be processed close to where data are generated, i.e., shifting cloud computing capabilities to the network edge such as roadside units (RSUs), enabling localized and real-time processing for intelligent transportation systems [13,14].

Numerous approaches and frameworks have been recently developed for implementing computational offloading in vehicular edge cloud computing (VECC) systems [15,16]. From one perspective, some models focus on a singular objective, while others cover multi-objectives [17]. From another perspective, several approaches address task offloading for a singular-edge server, while others serve multi-edge servers, with or without cloud integration [18]. While existing task offloading models address both single and multi-objective optimization (e.g., [17]) and single and multi-edge server deployments with or without cloud integration (e.g., [18]), the majority of current solutions permit vehicles to transfer their computational tasks only to connected RSUs or edge servers, resulting in imbalanced workloads at these stations [19,20]. Consequently, in dynamic or congested environments, individual vehicles may struggle to meet latency constraints for task completion. Moreover, optimizing task offloading strategies for multiple vehicles in complex, dynamic systems, such as multi-user VEC systems, remains a significant challenge that requires careful consideration. Furthermore, VECC failure cases in recent years have been attributed to uneven load distribution and security vulnerabilities [12,21]. To address these challenges, we present an efficient framework for VECC networks to distribute the workload among RSUs and minimize energy consumption evenly. Furthermore, we implement a robust security layer to safeguard crucial information during transmission. A novel caching approach is further implemented to enhance energy efficiency in-vehicle networks. The key contributions of this work are detailed below:

- An optimized load-balancing technique is presented to enhance the load distribution among RSUs, wherein vehicles are reassigned to the most suitable RSUs according to their task size, location, and CPU cycles. Moreover, UAVs are employed alongside edge servers to deliver computational and communicative resources by hovering over congested areas where the RSU servers remain overwhelmed.
- A robust security layer is developed, combining AES with dynamic one-time encryption key generation to secure data transfer, ensuring improved critical information security during offloading.
- A new caching technique for edge servers is employed, concentrating on the selective caching of application code and task-specific data to minimize energy consumption while maintaining latency requirements. The caching strategy considers server capacity, task popularity, and data size to enhance efficiency and reduce energy consumption.
- A comprehensive model is developed that integrates computation offloading, security, load balancing, and task caching, aiming to minimize energy consumption in vehicles while meeting the latency demands.
- Given the problem’s NP-hard complexity, the paper develops a deep learning-based algorithm and an equivalent reinforcement learning model to solve it efficiently, allowing for effective decision making in dynamic environments.
- Simulation results proved that the proposed model demonstrates fast and efficient convergence while significantly outperforming current benchmark techniques in reducing system energy consumption.

The remainder sections of this study are structured as follows: Section 2 summarizes existing research on task offloading models. Section 3 introduces the system model and formulates the problem. Section 4 subsequently details the formulation of an efficient computation offloading algorithm that employs deep learning to find the close-optimal solution. The experimental findings are then given and analyzed in Section 5. Finally, Section 6 concludes the study and provides suggestions for future research.

## 2. Related Work

Computation offloading has been gradually researched to optimize the challenges faced by vehicles through using VECC networks. Diverse methodologies and optimization frameworks have been developed employing traditional techniques [9,16], while deep learning-based techniques have recently been adopted to address this problem [22,23]. This section provides a concise overview of prevalent models, encompassing both traditional and deep learning-based approaches. Table 1 provides a comprehensive summary of the reviewed literature, outlining their objectives, proposed methodologies, security considerations, and key limitations.

### 2.1. Traditional-Based Techniques

Wu et al. have developed a formulation for a many-objective computation offloading problem within the hierarchical VEC network to optimize task completion time, energy consumption, resource costs, and load balance [24]. Then, a bio-inspired optimization algorithm based on Invasive Tumor Growth Optimization is proposed, where four types of tumor cells with different search strategies are employed to boost the effectiveness of the search. This algorithm integrates dimension-based coarse-grained and fine-grained search policies and a density screening rule to enhance efficiency and accuracy. Nevertheless, a significant limitation of this approach is its difficulty in integrating various VEC properties and search algorithms while maintaining high efficiency. In addition, this approach does not address security concerns related to transmitting sensitive data during offloading.

An efficient and secure multi-user multi-task computation offloading model is proposed in [25] to enhance MEC in mobile IoT networks. They formulate an integer nonlinear optimization problem to minimize the weighted sum of energy consumption under latency constraints. To achieve this, they integrate resource allocation, compression, and security mechanisms, employing JPEG and MPEG4 algorithms to reduce data transfer overhead. Additionally, a security layer is incorporated to protect data from cyberattacks. To ensure that the NP-hard nature of the optimization problem, linearization, and relaxation techniques are utilized, transforming it into a convex form. Finally, a multi-user multi-task computation offloading algorithm is designed to provide near-optimal task offloading decisions, significantly improving system efficiency and scalability. However, the approach lacks efficient load balancing among edge node servers, impacting performance and resource utilization. Additionally, task caching is not considered, which could further optimize computation offloading efficiency. Meanwhile, an innovative UAV-assisted vehicular computation offloading framework is proposed in [26] to address the overload problem in VEC networks. They formulate an online optimization problem to reduce vehicular task delays and accommodate the UAVs’ long-term energy sustainability. Afterward, the Lyapunov optimization technique is employed to separate the energy constraints and enable immediate problem solving without prior information. Further, the framework adopts a Markov approximation strategy to derive a nearly optimal performance level that closely aligns with the theoretical solution. Nonetheless, the framework does not consider the specific nature of computational tasks when distributing resources nor addresses the security concerns associated with transmitting sensitive information during offloading.

Recently, an energy-efficient and security-aware task offloading framework is proposed in [27] to optimize task execution in multi-tier edge-cloud computing systems. An optimization problem is also formulated to minimize mobile devices’ energy consumption while ensuring data security. To achieve this, an Advanced Encryption Standard (AES)-based cryptographic method combined with fingerprint authentication to secure data transmission during offloading is introduced. Further, a novel load-balancing algorithm is developed to redistribute tasks among edge servers based on location, CPU cycles, user load, and available bandwidth. However, this approach lacks efficient load balancing for overloaded edge nodes outside vehicle intersection areas and ignores task caching, which could significantly reduce mobile energy consumption. Meanwhile, Yuan et al. recently introduced a cost-effective computation offloading framework designed for UAV-assisted MEC environments, incorporating a multi-tiered UAV network with cloud-edge capabilities to accommodate multiple mobile users [28]. They formulated a mixed-integer nonlinear model focused on reducing the total costs associated with latency and energy while enhancing caching processes and allocating computational resources. Finally, a novel hybrid metaheuristic-based algorithm was developed to resolve this problem effectively and significantly, improving the system’s energy efficiency and latency performance.

### 2.2. Deep Learning-Based Techniques

Deep learning techniques have witnessed extensive deployment across various domains, including computer vision, natural language processing, gaming, and speech recognition, as detailed in recent literature [41]. In particular, applications of reinforcement learning paradigms have been explored in a few recent studies on vehicular edge computing systems [42,43], demonstrating its efficacy in empirically addressing complex, large-scale challenges. For instance, a deep reinforcement learning (DRL)-based approach was proposed for computation offloading in vehicular edge computing networks [29]. Specifically, the idle vehicular resources alongside MEC servers were utilized as computational edge resources to effectively handle computation-intensive and latency-sensitive tasks, aiming to manage computation-intensive and latency-sensitive tasks efficiently. In addition, this approach incorporates a multi-agent DRL architecture with an advanced actor-critic network and utilizes a combination of prioritized experience replay and adaptive n-step learning to augment both efficiency and overall performance. Meanwhile, Zhao et al. [30] propose a digital twin-assisted intelligent partial offloading scheme for VEC environments, integrating an improved clustering algorithm, Deep Reinforcement Learning (DRL), and a novel feedback mechanism. The approach optimizes the offloading decision space by reducing complexity through clustering and dynamically adjusts computational delay and vehicle service cost using DRL. Additionally, the feedback mechanism enhances coordination between digital and physical spaces by refining clustering parameters based on offloading outcomes. Extensive experiments demonstrate that the approach significantly reduces total computational delay, improves offloading success rates, and lowers overall system costs compared with existing methods. Nevertheless, [29] identifies a significant limitation regarding the complexity of the training process, where a large state space and variable temporal parameters present challenges in developing effective offloading techniques with solely fully connected networks. Meanwhile, security and task caching issues are not considered in [30].

Similar to the enumerated efforts, Liu et al. [31] introduced a novel framework for multi-user computation offloading and a resource allocation framework for a vehicular edge network, employing the deep deterministic policy gradient algorithm. This framework models the problem as a mixed-integer nonlinear programming task, aiming to minimize total system delay by effectively managing the large state spaces and mixed-variable action spaces. The results demonstrated that this approach not only enhances the quality of service in task execution but also proves stability and scalability in simulations. Meanwhile, a security-aware task offloading framework using deep reinforcement learning is proposed in [32] to address security and efficiency challenges in MEC systems. They formulate an optimization problem to minimize task execution latency and energy consumption while ensuring data security. Subsequently, a Markov Decision Process is employed to model the system utility, enabling efficient decision making for offloading tasks. Furthermore, a Proximal Policy Optimization (PPO)-based deep reinforcement learning algorithm is designed to derive optimal offloading strategies dynamically. Furthermore, Tian et al. presents a joint task caching and computation offloading scheme for vehicular edge computing using Deep Reinforcement Learning [33]. They propose a vehicle-edge-cloud architecture to optimize resource utilization among vehicles, edge servers, and cloud infrastructure. By utilizing a deep deterministic policy gradient, the method dynamically decides task caching and offloading strategies to minimize task processing latency and energy consumption. The proposed scheme efficiently reduces computational overhead by utilizing partial offloading and collaboration among edge servers. Meanwhile, in [34], a novel double deep Q-network-based approach with dynamic offloading is proposed within mobile edge computing systems. This model is tailored to manage offloading tasks in environments where servers are organized in a regular hexagonal structure. This design optimizes service latency across a network, improving load distribution and minimizing transmission delays. The proposed model effectively manages the computational demands by optimizing task distribution among edge servers, thereby enhancing the overall efficiency and responsiveness of the network. However, a significant limitation of these works is their disregard for security concerns, particularly the risks associated with transmitting sensitive information during the offloading process, which is recognized as a critical issue.

Recent advances in computation offloading models for vehicular edge computing have been detailed in the literature [35,36,37,38]. Specifically, Xue et al. [35] presented a multi-agent deep reinforcement learning framework that includes a novel task migration strategy to ensure service continuity as vehicles leave the range of Road-Side Units (RSUs), significantly reducing computational overhead. Conversely, in [36], digital twin technology is employed for real-time data acquisition and utilizes a spatiotemporal graph neural network for demand forecasting alongside an enhanced A3C algorithm for task caching. This integration markedly diminishes unnecessary computations and delays. Furthermore, Yang et al. [37] introduced a service-aware offloading strategy that leverages real-world vehicular data for dynamic service prediction. This strategy allows RSUs to pre-cache services, facilitating informed offloading decisions by vehicles and notably reducing task processing delays through adaptations to fluctuating service demands, thus enhancing computational efficiency in vehicular edge computing (VEC). In their contribution, Min et al. presented a detailed framework for high-mobility VECC networks [38]. The authors developed a model integrating multi-task offloading with resource allocation management. This model effectively utilizes task prioritization, context-aware decision making, multi-agent collaboration, and distributed learning to minimize energy, communication, and computational expenses. Subsequently, the double deep Q-network-based algorithm is designed to solve this problem efficiently, handle the overestimation issues in the Q-learning technique, and improve the converge performance. Following this, they developed an algorithm based on the double deep Q-network to efficiently solve this problem, mitigate overestimation biases commonly found in traditional Q-learning methods, and enhance convergence performance. However, despite these advancements, these studies do not adequately address the need for load balancing among edge node servers. Moreover, a critical shortcoming of some of these models is their insufficient attention to security, particularly the risks associated with transmitting sensitive information during offloading processes.

Furthermore, an innovative federated learning with a blockchain-based task scheduling framework is proposed in [40] to enhance security and efficiency in mobile cloud computing. The study formulates an optimization problem for computational offloading while ensuring data privacy and integrity. Subsequently, the Federated Learning with Blockchain Technology approach is introduced to enable distributed model training without sharing raw data, thereby enhancing privacy protection. Moreover, an Optimization Task scheduling-based Computational Offloading framework is designed to efficiently match resources, sequence tasks, and optimize scheduling in microservices-based MCC applications. Finally, the Adaptive Salp Swarm Algorithm is employed to improve task execution efficiency, minimize computational costs, and enhance Quality of Service. Meanwhile, a federated learning and blockchain-enabled framework is proposed in [39] to enhance traffic rerouting and task offloading in the Internet of Vehicles (IoV). The study optimizes traffic congestion, data security, and computational resource allocation in edge-cloud environments. Federated learning ensures privacy-preserving model training, while blockchain guarantees data integrity and trust. An efficient task offloading strategy dynamically distributes computational loads, reducing latency and energy consumption. Additionally, a hybrid traffic rerouting algorithm, combining Ant Colony Optimization and Deep Reinforcement Learning (DRL), optimizes vehicle routes in real time.

It is observed from the above literature review that computation offloading has addressed various challenges for the VECC environment through different scenarios and solver techniques. However, significant security issues during data transmission are still not well considered. Additionally, while there are existing strategies involving caching and delivery of content and tasks to reduce transmission loads on networks, task caching is not as extensively explored as content caching. Moreover, most existing studies fail to effectively manage workload distribution across different edge server settings, which could lead to increased energy consumption in vehicles. Further, formulating an effective policy for these dynamic and time-sensitive environments remains a persistent challenge. In response to these gaps, our study proposes a robust framework for VECC that optimizes workload distribution across RSUs, enhances data security during transmission, and minimizes energy consumption in vehicular environments.

## 3. System Model

This section begins by presenting the foundational components of the VECC environment, including the network, load balancing, communication, and computation models. Subsequently, a detailed discussion of the security strategies and the proposed caching mechanism is provided. Finally, the formulation of the optimization problem is addressed, with a primary focus on minimizing the overall energy cost of the system.

### 3.1. Network Model

The system architecture is organized into three main tiers. The initial tier comprises a set of vehicles, represented as V={1,…,M}, each assigned to perform a set of computationally demanding tasks Q={1,…,N}. The second tier includes two key components: a set of roadside units (RSUs) G={1,…,K} and a set of unmanned aerial vehicles (UAVs) U={1,…,U}. These entities provide essential storage and computing resources to assist vehicles. In particular, UAVs are used as moving servers that improve communication and computational resource allocation by strategically hovering over areas of high density, thereby complementing the fixed positions of pre-deployed RSUs. Furthermore, Software Defined Networking (SDN) controllers orchestrate the operations of both RSUs and UAVs, guaranteeing effective resource management. The third tier consists of a centralized cloud infrastructure linked to the SDN controllers via the network core.

**Figure 1:**
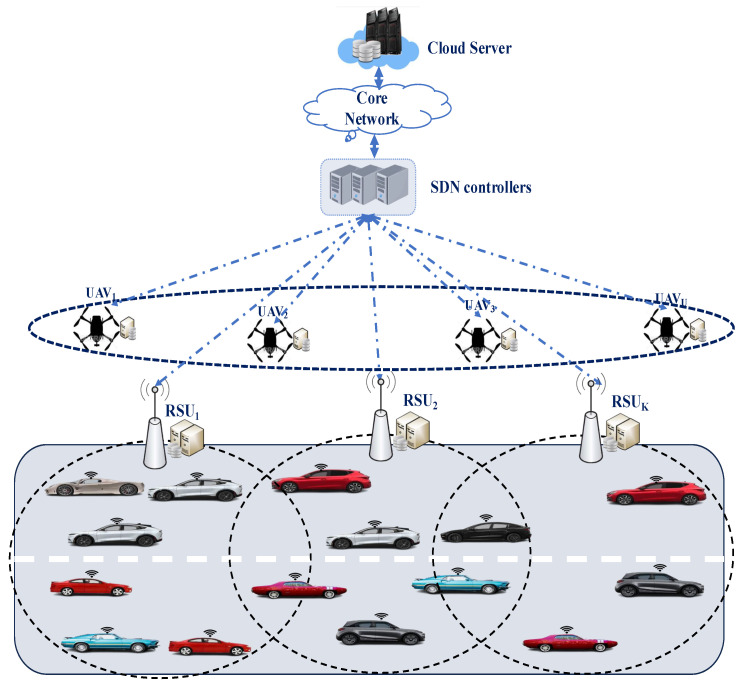
System model.

Consequently, depending on the environment, each vehicle’s computational tasks can be either dynamically processed locally or offloaded to one of the available servers. To formally represent this, the set of available servers is denoted as S={0,1,…,K,K+1,K+2,…,U,U+1}, where 0 signifies local processing on the vehicle itself, 1 to *K* correspond to the available RSUs, K+1 to *U* represent the available UAVs, and U+1 denotes remote execution on the cloud (see Figure 1). Moreover, to formalize the offloading decision, let $\alpha_{ijk}\in \left\{0,1\right\}$ signify the offloading of task *j* from vehicle *i* to server *k* for processing. Specifically, ($\alpha_{ij0}=1$) denotes local execution on the vehicle itself, while ($\alpha_{ijU+1}=1$) indicates cloud-based processing. Otherwise, the task from vehicle i is offloaded and processed remotely on one of the available RSUs or UAVs. Ensuring that each task is executed exclusively on a single server, locally, at the edge, or in the cloud is crucial. To achieve this, we enforce the following constraint:

(1) $$\sum_{k=1}^{U+1}\alpha_{ijk}=1$$

### 3.2. Load Balancing

**Figure 2:**
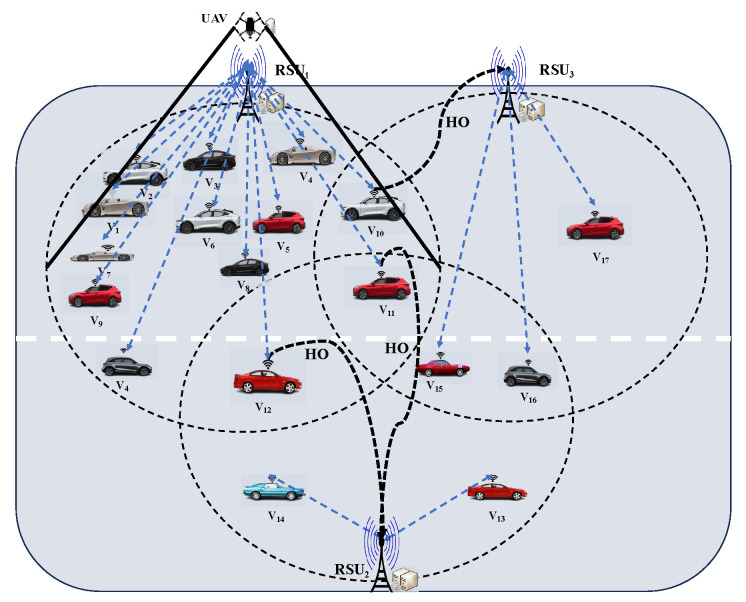
Snapshot of vehicles’ distribution scenario within the network.

This subsection outlines the design of a load-balancing mechanism for vehicles among RSUs utilizing the assistance of UAVs. Figure 2 presents a snapshot of the vehicle distribution scenario at a given time *t*, highlighting the imbalance in vehicle allocation across RSUs. This imbalance leads to network congestion, significantly degrading service quality and increasing application latency for connected vehicles. To address this issue, this study proposes a load-balancing mechanism to achieve equitable resource utilization across all RSUs. This objective is achieved through a two-phase approach. In the initial phase, vehicles situated in the overlapping areas of RSUs are strategically reassigned to connect with the least loaded RSUs within their coverage range by initiating a handover process. In the subsequent phase, the system detects RSUs that remain overloaded beyond a predefined threshold value (ι) and mitigates their burden by deploying UAVs to hover above them, offering supplementary computational and storage resources for their associated vehicles. The detailed steps involved in implementing this load-balancing mechanism are described as follows:

The SDN controller first receives a summary of information regarding the vehicles linked to each RSU through the interconnected RSUs. This information includes the vehicles’ number, the data rates accessible for each vehicle, cycles of CPU and data necessary for each task, and details regarding vehicles situated in overlapping coverage areas of RSUs that may be reassigned to different RSUs. The SDN controller systematically assesses each vehicle in the overlapping coverage zones and dynamically reallocates them to the least loaded RSU within their coverage area by initiating a handover process. After this reassignment, the controller modifies each vehicle’s computational capabilities and data rates per its new association with the specified RSU. The iterative process continues until the optimal assignment of all vehicles to RSUs is achieved. Subsequently, the controller re-evaluates the load distribution across all RSUs and identifies those that remain overloaded where the number of connected vehicles exceeds a predefined threshold (ι). Subsequently, the controller deploys one or more UAVs to hover above, enhancing the computational and storage capabilities of the overloaded RSUs. This approach thereby effectively minimizes the overhead for vehicles and improves system efficiency. Algorithm 1 provides a detailed outline of the complete load-balancing process.

In this example, shown in Figure 2, we observe that 17 vehicles are distributed among three RSUs, with 12 vehicles connected to $RSU_{1}$, while $RSU_{2}\;RSU_{3}$ are connected to 2 and 3 vehicles, respectively. Notably, vehicles $V_{10}$, $V_{11}$, $V_{12}$, $V_{15}$, and $V_{16}$ are positioned near RSU boundaries, making their reallocation to other RSUs more feasible. Moreover, each RSU provides computational resources of 20 GHz and a bandwidth of 20 MHz, which is shared among connected vehicles. The major objective is to reallocate workloads among RSUs to improve service quality and decrease energy consumption while also employing UAV-enabled edge computing for RSUs that are persistently overcrowded. As mentioned above, this redistribution process is executed in two key phases, where vehicles are first reassigned to the most suitable RSU through a handover mechanism. Afterward, UAVs are deployed to provide additional computational support for RSUs that exceed their capacity thresholds. The steps required to achieve these goals are outlined based on the specified parameters. Using the parameter values summarized in Table 2, these phases can be effectively implemented as follows:
**Algorithm 1** Optimizing RSUs’ Load Distribution  1:**Initialization**: Each vehicle *i* is linked with a corresponding RSU *k*.  2:/*Initial Phase—Reallocate vehicles across RSUs*/  3:**for all** RSUs *k* during the current time slot *t* **do**  4: μ← Total number of vehicles connected to each RSU *k*  5: λ← Computational requirements of connected vehicles, including CPU cycles and task data sizes.  6: ψ← Compute the available computational resources and data rates allocated for each vehicle at RSU *k*.  7: ℵ← Identify vehicles located within RSU overlapping zones that can be reassigned to nearby RSUs.  8: For each identified vehicle, determine the optimal RSU with the least load by considering μ, λ, and ψ, and initiate the handover process.  9:**end for**10:/*Second Phase—Deploy UAVs for Overloaded RSUs*/11:**for all** RSUs *j* during the current time slot *t* **do**12: μ← Updated number of vehicles connected to RSU *k*.13: **if** μ > ι **then**14:  Deploy a UAV with sufficient computational and storage capabilities to hover above RSU *k* and alleviate its load.15: **end if**16:**end for**

Table 2 presents a summary of the initial system state, including the number of RSUs, their associated vehicles, and key vehicle parameters such as available data rates, task requirements (data size in MB), and CPU cycles in Gigacycles), and potential for reallocation. The SDN controller then iteratively analyzes each vehicle within the overlapping coverage areas. The controller determines the optimal RSU for each vehicle based on predicted processing time, encompassing both transmission and computation delays. For instance, vehicle $V_{10}$ is optimally reassigned to $RSU_{3}$, where the predicted processing time is 6.8 s, compared with 23.2 s at $RSU_{1}$. Similarly, vehicles $V_{11}$ and $V_{12}$ are handed over to $RSU_{2}$ to minimize processing time, while vehicles $V_{15}$ and $V_{16}$ remain connected to $RSU_{3}$. Following this initial phase of vehicle reassignment, the SDN controller re-evaluates the load distribution across all RSUs and identifies the RSUs that have connected vehicle count, which exceeds a predefined threshold (e.g., ι = 6), which is $RSU_{1}$ in this example. Subsequently, a UAV is deployed over $RSU_{1}$ to provide additional computational resources, thereby enhancing overall system performance and minimizing communication overhead.

### 3.3. Communication Model

This subsection introduces the energy consumption and transmission time involved in communication links between the vehicles and the servers. Additionally, each computationally intensive task is characterized by a tuple $(\beta_{ij},\sigma_{ij}$, and $\delta_{ij}$, where i∈M and j∈N. Here, $(\beta_{ij},\sigma_{ij}$, $\delta_{ij})$ correspond to the input data size, output data size, and computational demand in CPU cycles required for task *j* of vehicle *i*, respectively. Moreover, following the approach outlined in [44], this study disregards the energy and time consumption associated with transmitting output data. This decision is justified by the relatively small size of the output data compared with the input data, rendering its impact negligible in the overall analysis. Additionally, informed by the insights from [45,46], this study utilizes a quasi-static model for simulation, wherein the number of vehicles remains fixed during an individual offloading period but can fluctuate across successive offloading periods.

Furthermore, inspired by the results of [47], this study adopts an orthogonal frequency division multiple access (OFDMA) strategy to enable simultaneous task offloading over the same channel, thereby mitigating intra-cell interference. Building upon the foundational principles of Shannon’s channel capacity theorem, the upload data rate between a vehicle *i* and the edge server (RSUs or UAVs) for task *j* can be mathematically expressed as follows:

(2) $$R_{ik}=B_{ik}log_{2}\left(1+\frac{p_{i}g^{2}}{\omega B_{ik}}\right)$$

where $B_{ik}$ represents the uplink bandwidth allocated to the vehicle, while $p_{i}$ indicates the vehicle’s transmission power. Furthermore, the parameters *g* and ω correspond to the channel gain and the noise power at the edge server, respectively.

### 3.4. Computation Model

This subsection outlines the computational model designed for our environment, consisting of *K* sets of RSUs, *U* sets of UAVs, and *M* sets of vehicles, each linked to *N* sets of computationally intensive tasks. The tasks may be executed locally within the vehicles or offloaded to an edge server (RSU or UAV) or a cloud server for processing. The following subsections analyze this framework’s local and remote computing processes in detail.

#### 3.4.1. Local Processing

This study recognizes the heterogeneity in processing capabilities among different vehicles. Consequently, the energy and time required for local task execution can be accurately quantified and estimated as follows:

(3) $$E_{ij}^{L}=\delta_{ij}\eta_{i}$$

(4) $$T_{ij}^{L}=\frac{\delta_{ij}}{f_{i}^{L}}$$

where $\eta_{i}$ represents the energy consumed per CPU cycle and $f_{i}^{L}$ denotes the processing capacity of vehicle *i*.

#### 3.4.2. Remote Processing

The execution of vehicle tasks on remote servers, such as RSUs, UAVs, or the cloud, is investigated in this subsection. Thus, it is possible to precisely estimate the energy usage and time needed by the servers as follows:

(5) $$E_{ij}^{R}=p_{i}T_{ij}^{Tran}$$

(6) $$T_{ij}^{RSU}=T_{ij}^{Tran}+T_{ij}^{RSU\_ex}$$

(7) $$T_{ij}^{UAV}=T_{ij}^{Tran}+T_{ij}^{UAV\_ex}$$

(8) $$T_{ij}^{CLO}=T_{ij}^{Tran}+\varpi +T_{ij}^{CLO\_ex}$$

where $T_{ij}^{Tran}$, $T_{ij}^{RSU\_ex}$, and $T_{ij}^{UAV\_ex}$ respectively denote the task transmission time, RSU execution time, and UAV execution time, which can be accurately quantified and estimated using Equations (9)–(11). Additionally, $T_{ij}^{CLO\_ex}$ and ϖ represent the cloud execution time and the propagation delay between the edge server (RSU or UAV) and the cloud server, which can be accurately quantified and estimated using Equation (12).

(9) $$T_{ij}^{Tran}=\frac{\beta_{ij}}{R_{ik}}$$

(10) $$T_{ij}^{RSU\_ex}=\frac{\delta_{ij}}{f_{i}^{RSU}}$$

(11) $$T_{ij}^{UAV\_ex}=\frac{\delta_{ij}}{f_{i}^{UAV}}$$

(12) $$T_{ij}^{CLO\_ex}=\frac{\delta_{ij}}{f_{i}^{CLO}}$$

Moreover, $f_{i}^{RSU}$, $f_{i}^{UAV}$, and $f_{i}^{CLO}$ respectively denote the processing capacities available to vehicle *i* at the RSU, UAV, and Cloud server.

It should be noted that the total computing resources of the RSU and UAV servers are dynamically distributed to linked vehicles and are denoted by $Fc^{RSU}$ and $F_{c}^{UAV}$, respectively. To ensure efficient resource utilization and prevent system overload, it is important to consider the following two constraints:

(13) $$\sum_{i=1}^{M}\sum_{j=1}^{N}\sum_{k=1}^{K}\alpha_{ijk}f_{i}^{RSU}\leq F_{c}^{RSU}$$

(14) $$\sum_{i=1}^{M}\sum_{j=1}^{N}\sum_{k=K+1}^{U}\alpha_{ijk}f_{i}^{UAV}\leq F_{c}^{UAV}$$

### 3.5. Security

Using wireless channels to transmit application data from vehicles to VECC servers introduces significant security risks, including cyberattacks and potential exposure of sensitive information [21]. To mitigate these risks and enhance data protection against brute-force attacks, this subsection presents a robust security layer that integrates the Advanced Encryption Standard (AES) algorithm [48] with a dynamic key generation mechanism. Specifically, we employ a one-time encryption key generation technique [49] that ensures that each communication session is secured with a unique key, thereby reducing susceptibility to key compromise.

While AES is widely recognized for its efficiency and security, its structured encryption approach makes it susceptible to cryptographic attacks due to uniform block encryption and algebraic properties. We propose an enhanced key generation process within the AES framework to address these vulnerabilities, utilizing a dynamic and unpredictable one-time encryption technique. This approach generates highly randomized keys per session, preventing replay attacks and reducing the risk of key reuse. Furthermore, we incorporate a secure key distribution protocol to facilitate efficient and authenticated key exchange between vehicles and VECC servers. This protocol integrates mutual authentication mechanisms to verify communicating entities and prevent man-in-the-middle attacks. Additionally, to counter replay attacks, our method employs timestamp-based validation and nonce-based verification, ensuring that each communication instance remains unique and cannot be maliciously replayed. By strengthening the randomness and unpredictability of key generation while securing distribution and authentication, our approach significantly enhances the overall security and resilience of the encryption process [25,27].

Subsequently, $\tau_{ij}\in \left\{0,1\right\}$ indicates a binary decision variable representing security decisions. In particular, $\tau_{ij}=0$ signifies that the task *j* is considered non-sensitive, negating the need for encryption. On the other hand, $\tau_{ij}=1$ indicates that the security layer has encrypted the task data. This framework allows for user-specific security preferences, enabling individuals to personalize their security settings according to their distinct data privacy needs. Moreover, implementing this security layer may result in increased processing and energy overhead. This overhead can be defined as follows:

(15) $$e_{ij}^{ENC}=ENC_{ij}\eta_{i}$$

(16) $$t_{ij}^{ENC\_DEC\_RSU}=\frac{ENC_{ij}}{f_{i}^{L}}+\frac{DEC_{ij}}{f_{i}^{RSU}}$$

(17) $$t_{ij}^{ENC\_DEC\_UAV}=\frac{ENC_{ij}}{f_{i}^{L}}+\frac{DEC_{ij}}{f_{i}^{UAV}}$$

where $ENC_{ij}$ and $DEC_{ij}$ represent the processing cycles required to execute encryption and decryption operations, respectively, for task *j* of vehicle *i* and the corresponding server (RSU or UAV). Moreover, considering the security decisions made for each task, the total communication overhead incurred during the transmission can be accurately expressed as follows:

(18) $$E_{ij}^{SEC}=\left[\tau_{i}\left(e_{ij}^{ENC}+E_{ij}^{R}\right)+\left(1-\tau_{i}\right)E_{ijk}^{R}\right]$$

(19) $$\begin{array}{cc} T_{ij}^{SEC} & =\sum_{k=1}^{K}\left[\tau_{i}\left(t_{ij}^{ENC\_DEC\_RSU}+T_{ij}^{RSU}\right)+\left(1-\tau_{i}\right)T_{ij}^{RSU}\right]\\ \; & +\sum_{k=K+1}^{U}\left[\tau_{i}\left(t_{ij}^{ENC\_DEC\_UAV}+T_{ij}^{UAV}\right)+\left(1-\tau_{i}\right)T_{ij}^{UAV}\right] \end{array}$$

### 3.6. Task Caching

The task caching mechanism used in the edge server is investigated in this study, emphasizing caching previously completed tasks and the associated data for possible future use. According to earlier studies, this strategy prioritizes caching decisions by considering computational demand, data size, and request frequency [50].

The task caching mechanism works as follows: The edge server carefully gathers detailed information, including the computation tasks and their frequency of requests. An optimum caching strategy is established to reduce the vehicle network’s energy use. The vehicle initiates the offloading of computational tasks to the edge server. When a specific task is not cached, the relevant program and associated data are sent to the edge server for processing. If the task has been previously cached, the server processes it directly and returns the results to the vehicle without delay. This caching mechanism reduces vehicles’ need to repeatedly offload identical tasks, significantly decreasing the total time and energy costs related to task execution.

To properly control the caching mechanism, a binary decision variable $\upsilon_{ij}\in \left\{0,1\right\}$ is established. Here, $\upsilon_{ij}=1$ indicates that task *j* from vehicle *i* has been cached at the edge server. Otherwise, the task is still uncached and must be sent to be processed. $F_{s}^{RSU}$ and $F_{s}^{UAV}$, respectively, reflect the cache storage capacity of the RSU and UAV servers. The following two restrictions must be taken into account to guarantee effective resource use and avoid system overload, as follows:

(20) $$\sum_{i=1}^{M}\sum_{j=1}^{N}\upsilon_{ij}f_{i}^{RSU}\leq F_{s}^{RSU}$$

(21) $$\sum_{i=1}^{M}\sum_{j=1}^{N}\upsilon_{ij}f_{i}^{UAV}\leq F_{s}^{UAV}$$

Finally, by incorporating offloading, load balancing, communication, computation, security, and task caching mechanisms, the total energy consumption and execution time required by vehicle *i* for process task *j* can be accurately expressed as follows:

(22) $$E_{ij}=\alpha_{ij0}E_{ij}^{L}+\sum_{k=1}^{U+1}\alpha_{ijk}\left(1-\upsilon_{ij}\right)E_{ij}^{SEC}$$

(23) $$\begin{array}{cc} T_{ij} & =\alpha_{ij0}T_{ij}^{L}+\sum_{k=1}^{K}\alpha_{ijk}\upsilon_{ij}T_{ij}^{RSU\_ex}+\left(1-\upsilon_{ij}\right)T_{ij}^{SEC}+\sum_{k=K+1}^{U}\alpha_{ijk}\upsilon_{ij}T_{ij}^{UAV\_ex}\\ \; & +\left(1-\upsilon_{ij}\right)T_{ij}^{SEC}+\alpha_{ijU+1}\upsilon_{ij}T_{ij}^{CLO\_ex}+\left(1-\upsilon_{ij}\right)T_{ij}^{SEC} \end{array}$$

### 3.7. Problem Formulation

This subsection presents a model for optimizing task offloading in multi-tiered VECC systems, ensuring the successful completion of all assigned tasks while minimizing energy consumption. The following is a detailed formulation of this constrained optimization problem:

(24) $$\begin{array}{cccccc} \; & \min_{\alpha } & \; & \sum_{i=1}^{M}\sum_{j=1}^{N}E_{ij} & \; & \\ \; & s.t. & \; & E_{ij}-E_{ij}^{L}\leq 0, & \; & C1\\ \; & \; & & T_{ij}-T_{ij}^{L}\leq 0, & \; & C2\\ \; & \; & & \sum_{k=0}^{K+1}\alpha_{ijk}=1, & \; & C3\\ \; & \; & & \sum_{i=1}^{M}\sum_{j=1}^{N}\upsilon_{ij}\beta_{ij}\leq F_{s}^{RSU}, & \; & C4\\ \; & \; & & \sum_{i=1}^{M}\sum_{j=1}^{N}\upsilon_{ij}f_{i}^{RSU}\leq F_{c}^{RSU}, & \; & C5\\ \; & \; & & \sum_{i=1}^{M}\sum_{j=1}^{N}\upsilon_{ij}\beta_{ij}\leq F_{s}^{UAV}, & \; & C6\\ \; & \; & & \sum_{i=1}^{M}\sum_{j=1}^{N}\upsilon_{ij}f_{i}^{UAV}\leq F_{c}^{UAV}, & \; & C7\\ \; & \; & & \alpha_{ijk}\in \left\{0,1\right\}, & \; & C8\\ \; & \; & & \tau_{ij}\in \left\{0,1\right\}, & \; & C9\\ \; & \; & & \upsilon_{ij}\in \left\{0,1\right\}, & \; & C10 \end{array}$$

Constraints C1 and C2 restrict energy consumption and task execution time, respectively, whereas constraint C3 guarantees that each task is performed only on one server. Additionally, the following four constraints are related to the computational resource limitations of the RSU and UAV servers. Furthermore, the last three constraints ensure that task offloading, security, and caching decisions are binary.

The optimal solution to the optimization problem formulated in Equation (24) necessitates the determination of optimal values for three key decision vectors: the computation offloading vector (α), the task security vector (τ), and the task caching vector (υ). However, the inherent non-convexity of the feasible region, compounded by the non-convexity of the objective function itself, presents a significant challenge. This complexity arises primarily from the binary nature of the decision variables α, τ, and υ. Consequently, the problem is classified as NP-hard [51], implying that finding an exact solution within polynomial time is computationally intractable. Furthermore, the complexity of the problem escalates significantly in a multi-tier VECC system due to the curse of dimensionality. Specifically, the problem’s computational complexity grows exponentially with an increase in the number of vehicles within the system. To circumvent these inherent challenges, we propose a novel approach that leverages the power of reinforcement deep learning. This innovative approach offers a more efficient and scalable alternative for determining close-optimal values for α, τ, and υ, thereby providing a viable solution to this complex optimization problem.

## 4. Proposed Distributed Deep Learning Algorithm

This section examines deep reinforcement learning to tackle and resolve complex optimization challenges proficiently. We briefly overview reinforcement learning, highlighting its fundamental ideas and essential elements. A new approach utilizing distributed deep Q-learning is introduced to obtain near-optimal solutions for the VECC system. This method employs deep learning to derive near-optimal policies for task offloading, security, and caching while alleviating the computational challenges inherent in conventional optimization techniques.

### 4.1. An Introduction to the Principles of Reinforcement Learning

**Figure 3:**
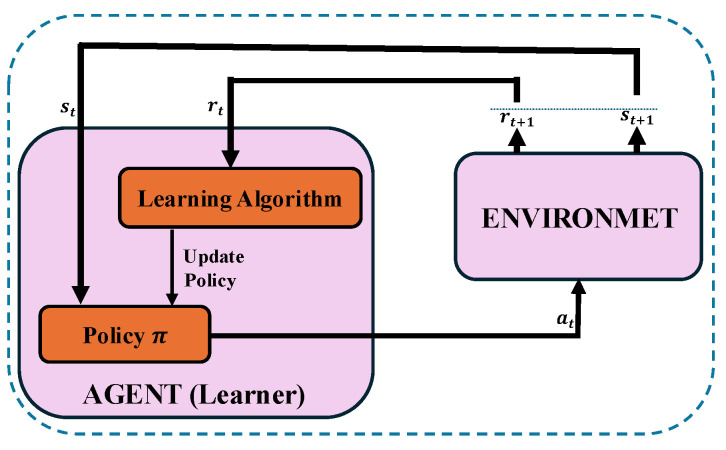
A graphical depiction of RL process.

Reinforcement learning (RL) is a fundamental branch of machine learning designed to address decision-making challenges in dynamic and uncertain environments [52]. The RL framework consists of five core components: the agent, the environment, the state, the action space, and the reward function, as depicted in Figure 3. At any given time step *t*, the agent perceives the current state of the environment, denoted as $s_{t}$. Then, based on this observation, the agent selects an action $a_{t}$ from a set of available actions, guided by a policy $\pi =(a_{t}|s_{t})$ that defines the likelihood of selecting a particular action given the current state. This action leads the agent to transition to a new state $s_{t+1}$ while yielding a reward $r_{t}$, determined by a reward function R(s,a). The primary objective of the RL agent is to learn a close-optimal policy that maximizes the expected cumulative reward $R_{t}=\sum_{i=0}^{\infty }\gamma^{i}r_{t+i}$ over an extended series of interactions with the environment, where γ∈[0,1] denotes a discount factor. Finally, this learning process involves continuous exploration and exploitation, enabling the agent to discover and exploit effective strategies for achieving its goals.

### 4.2. Essential Elements of the Reinforcement Learning Framework

To effectively represent the VECC system model within the reinforcement learning paradigm, it is crucial to define the mentioned key elements: state, action, and reward. Within the context of our multi-tier VECC environment, these elements can be described and characterized as follows:

- **State:** The state space, denoted by S, is defined by the essential task-specific attributes and, formally, at time *t*, can be represented as $s_{t}=\left\{{\left(\beta_{ij},\delta_{ij},R_{ik}\right)}_{t}\right\}$.
- **Action:** The action space, denoted by *A*, encompasses the caching and offloading decisions made by the system and, formally, at time *t*, can be represented as $a_{t}={\left(\alpha_{ijk},\upsilon_{ij}\right)}_{t}$, where the selection of each action is governed by a policy $\pi (a_{t}|s_{t})$.
- **Reward:** In this study, the objective function defined in Equation (24) is directly mapped to the reward function within the RL framework. Specifically, the reward $r_{t}$, at time *t*, is determined by evaluating the current state $s_{t}$ and the selected action $a_{t}$ according to the policy $\pi (a_{t}|s_{t})$. This process is repeated iteratively over time, where the cumulative reward $r_{t}$ is minimized according to the policy π, to optimize system performance through the formula $\lim_{T\to \infty }\frac{1}{T}\sum_{t=0}^{T}r_{t}$, where $r_{t}$ indicates the system energy $E_{ij}$ in Equation (24).

### 4.3. Robust Distributed Deep Reinforcement Learning

Distributed Deep RL (DDRL) is a sophisticated extension of the traditional Deep RL algorithm, designed to enhance decision-making efficiency through parallel computation, in which multiple deep neural networks (DNNs) are incorporated and simultaneously process the system states, generating Q-values for all possible actions and selecting the action associated with the minimal reward value [53]. This parallel framework not only accelerates the exploration of solution spaces but also improves computational performance. Leveraging the capabilities of DDRL, this study proposes a distributed deep RL-based algorithm to approximate the minimization of total reward values, effectively addressing the complexities of optimizing accumulative rewards in dynamic environments.

**Figure 4:**
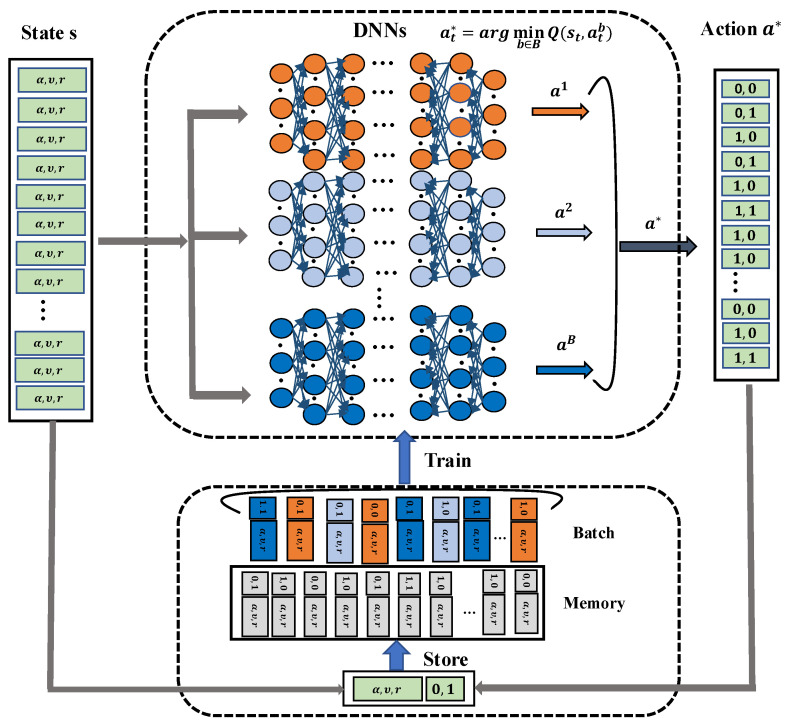
Proposed DDRL architecture.

Furthermore, Figure 4 illustrates the architecture of the proposed DDRL-based algorithm, which employs a set of DNNs, denoted by *B*, operating in parallel and connected to a shared replay memory *M* of fixed size. The process begins with the system receiving input data, comprising application task requirements and data rates (representing the system state). Each DNN processes this input independently, generating potential caching and offloading actions based on its current set of weights. These actions are then evaluated, and the action that minimizes the reward value is selected as the final output. This action is subsequently stored in the replay memory, contributing to the system’s learning experience. Over time, the DNNs are trained using randomly sampled data from the replay memory, allowing their weights to be updated iteratively. This training process enables the DNNs to refine their decision-making accuracy continually. Through this iterative learning mechanism, the model adapts and optimizes task offloading decisions, significantly enhancing the overall performance and efficiency of the system.

Subsequently, our proposed DDRL algorithm is outlined in Algorithm 2, where the caching and offloading decisions are effectively determined in an RL context. Specifically, the algorithm starts by initializing a set of *B* distinct DNNs with random weights $w_{t}^{b}$ and allocating a shared memory *M* with a fixed size *F*. Additionally, at each time *t*, the system takes the computational task requirements, including input size, CPU cycles, and data rates, as the input state $s_{t}$. Each DNN then independently processes these input states and generates a corresponding action $a_{t}^{b}$ (i.e., caching and offloading decision) based on the parameterized function $f_{w_{t}^{b}}:s_{t}\to a_{t}^{b}$. Among these actions, the one that minimizes the reward function is selected as the optimal action $a_{t}^{*}=arg\min_{b\in B}Q\left(s_{t},a_{t}^{b}\right)$. This chosen action is then stored in the replay memory *M* along with its corresponding state $s_{t}$ to contribute to the system’s learning experience. Further, a random mini-batch of stored transitions is sampled from the replay memory to ensure continuous improvement, and the DNN weights are updated based on these data. This iterative process enables the algorithm to adapt dynamically to changing system conditions, refine decision-making accuracy, and optimize caching and offloading over time. The DDRL algorithm ensures a scalable and efficient approach to addressing the challenges of task caching and offloading in distributed systems.
**Algorithm 2** Secured and Optimized DDRL Algorithm**Input:** Computational task demands $s_{t}$**Output:** Optimal caching and offloading decision $a_{t}^{*}$  1:Initialize each DNN with random weights $w_{t}^{b}$, where b∈B  2:Allocate memory *M* with a fixed size *F*.  3:**for **t=1,2,…,D **do**  4: Input the system state $s_{t}$ into each DNN.  5: Generate a set of candidate actions $a_{t}^{b}$ from the DNNs.  6: Select the optimal action $a_{t}^{*}$ that minimizes the reward value by utilizing $\arg\min_{b\in B}Q\left(s_{t},a_{t}^{b}\right)$  7: Store the transition $\left(s_{t},a_{t}^{*}\right)$ into the replay memory *M*.  8: Randomly sample a mini-batch of transitions from *M*  9: Train and update the weights of the DNNs based on the sampled transitions10:**end for**

## 5. Evaluation and Discussion of Simulation Results

This section begins by outlining the experimental setup in detail, followed by a comprehensive evaluation of the model’s performance through simulation-based analyses.

### 5.1. Simulation Setup

The simulation environment was designed with a scenario of a 150 m one-way road equipped with five randomly positioned RSUs and UAVs, each equipped to provide computational and storage capabilities. A total of 100 vehicles traversed this road, each carrying three computational tasks. The input data size for each task was randomly distributed within the [0,10] megabytes range, with a corresponding computational requirement of 500 CPU cycles/byte. A single cloud server was integrated into the system to augment computational resources and connect to the RSUs via an SDN controller. The cloud server was equipped with 500 GHz of processing power, while each RSU and UAV had 100 and 50 GHz. Vehicle computational resources were distributed within the [0.5,1.0] GHz range. All vehicles transmitted with a power of 100 mW, and each RSU was allocated a system bandwidth of 20 megahertz.

Moreover, this simulation was implemented using Python 3.7 on a personal computer featuring an Intel^®^ Core^™^ i7-4770 CPU operating at 2.4 GHz and 16 GB of RAM. The TensorFlow 2.15 and NumPy 2.1.3 libraries were employed to implement the deep-Q learning algorithm. The hyperparameters, including batch size, learning rate, and neural network architecture, were optimized through empirical experimentation, where different values were tested, and the best-performing configuration was selected. Specifically, the batch size and learning rate were tuned to ensure stable convergence and optimal learning efficiency. Regarding the neural network architecture, we adopted a two-layer structure with 120 and 80 neurons, respectively, based on the findings in [54], where a similar configuration demonstrated effective feature representation and decision-making capabilities in related tasks. This setup provided a balance between model complexity and performance, ensuring efficient learning within our system.

### 5.2. Experimental Results and Discussions

#### 5.2.1. Convergence Analysis

This subsection investigates the effect of applying different parameter values on convergence performance. Consequently, an in-depth analysis was conducted to determine the appropriate value for each parameter, which was subsequently adopted for all remaining simulations.

**Figure 5:**
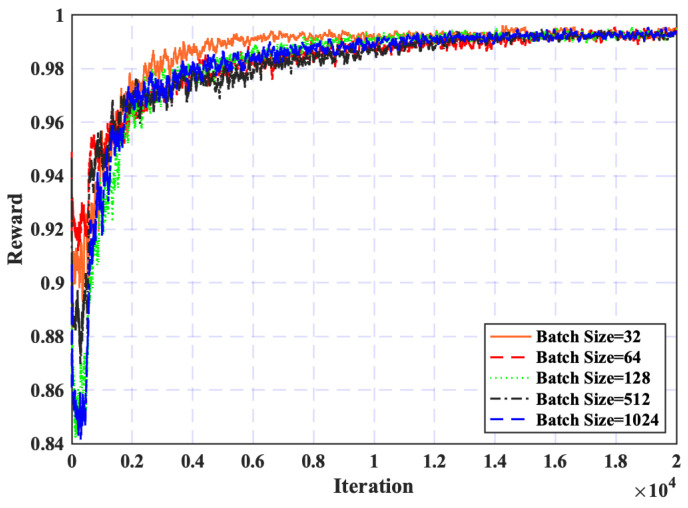
Batch size effect on convergence.

First, Figure 5 illustrates the impact of varying batch sizes on convergence performance. It is evident that a batch size of 32 achieves the most rapid convergence. This is attributed to the frequent updates of neural network weights enabled by the smaller mini-batch size, thereby accelerating the gradient descent process. However, with the larger values of batch sizes, such as 64, 128, 512, and 1024, they show slower convergence rates due to fewer frequent weight updates. Consequently, a batch size of 32 is identified as the most optimal choice for maximizing convergence efficiency and will be applied for subsequent simulations.

**Figure 6:**
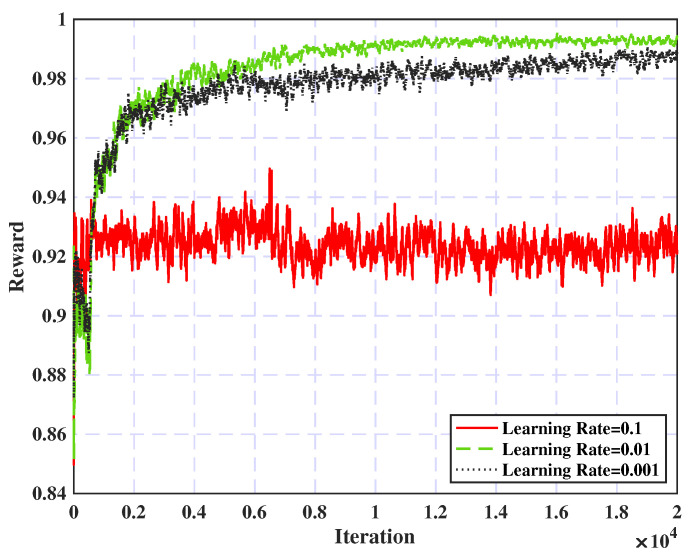
Learning rate effect on convergence.

Regarding the learning rate, shown in Figure 6, a learning rate of 0.01 demonstrates the most efficient convergence among the other values, achieving an optimal balance between speed and accuracy. In addition, higher learning rates (e.g., 0.1) initially accelerate convergence; however, they can ultimately decrease performance by dropping the model to local optima. On the other hand, lower learning rates (e.g., 0.001) may result in slower convergence. Consequently, a learning rate of 0.01 is the best choice and will be applied for subsequent simulations.

**Figure 7:**
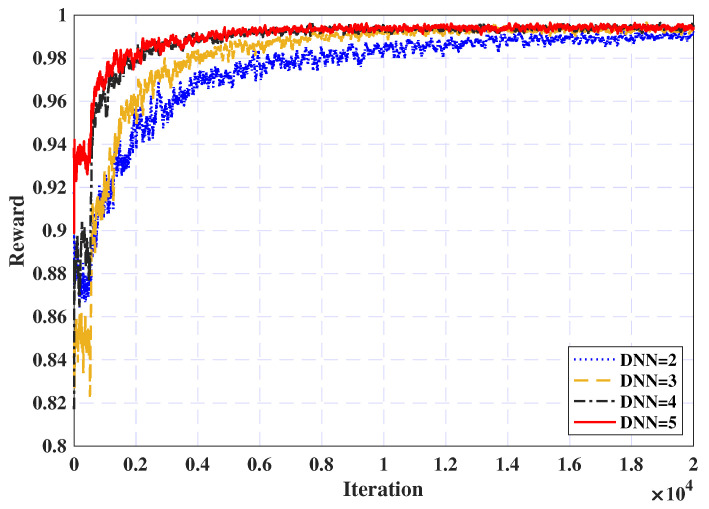
DNN effect on convergence.

Finally, Figure 7 illustrates the impact of varying the number of DNNs on convergence performance. It is evident from the figure that as the number of DNNs increases, the convergence accelerates, with the reward ratio improving significantly. Moreover, It is important to note that using three DNNs can achieve a 0.96 reward ratio within approximately 2000 iterations. In contrast, the model with fewer DNNs (e.g., DNNs = 1) exhibits slower convergence and reduced performance, highlighting the difficulty of reaching optimal convergence with lower network depths. Consequently, only three DNNs are identified as the best choice and will be applied for subsequent simulations.

#### 5.2.2. Task Caching Effect

**Figure 8:**
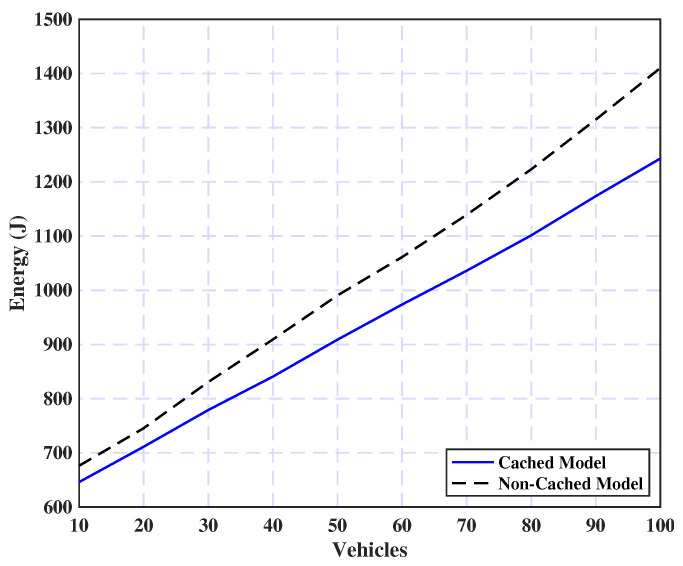
Performance impact of task caching on the model.

This subsection clearly illustrates the effect of integrating the proposed caching layer on our model, seen in Figure 8. It is evident from the figure that energy consumption rises proportionally with the number of vehicles for both the cached and non-cached models. However, the cached model consistently demonstrates superior energy efficiency compared with the non-cached model. This improvement is attributed to the caching mechanism’s ability to minimize redundant data transmissions and computational overhead, thereby reducing overall energy consumption, especially as the system scales and the number of vehicles grows.

#### 5.2.3. Load Balancing Effect

**Figure 9:**
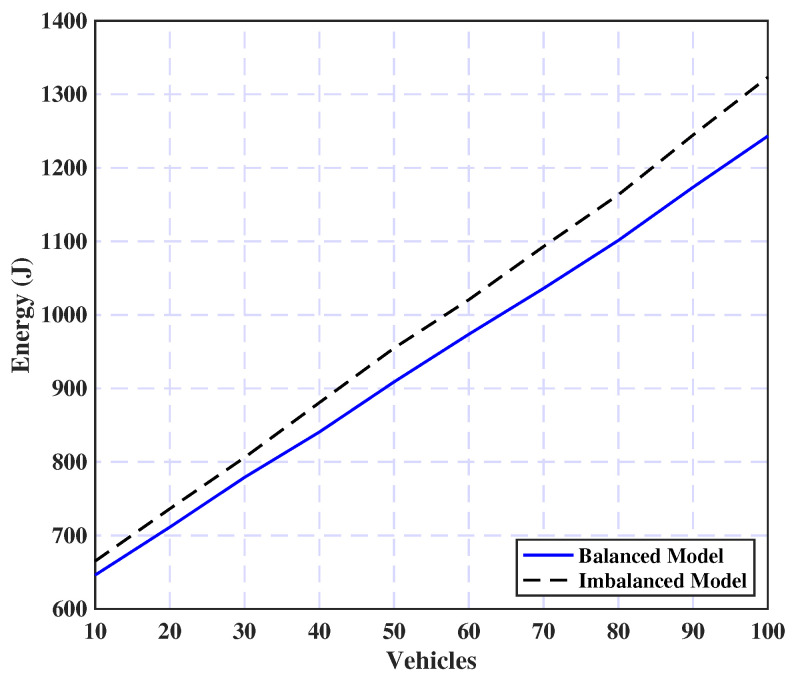
Performance impact of load balancing on the model.

This section evaluates the impact of implementing a load balancing algorithm (Algorithm 1) on our proposed model where the energy consumption is measured concerning the number of vehicles (see Figure 9). The results clearly show that both models, balanced and imbalanced, exhibit increased energy consumption with a growing number of vehicles. However, the balanced model consistently outperforms the imbalanced model in terms of energy efficiency. The efficiency gain is significantly increased as the number of vehicles exceeds approximately forty vehicles. This improvement is attributed to the algorithm’s ability to distribute computational tasks across vehicles efficiently, optimizing resource usage, especially as the system scales and the number of vehicles grows.

#### 5.2.4. Security Effect

**Figure 10:**
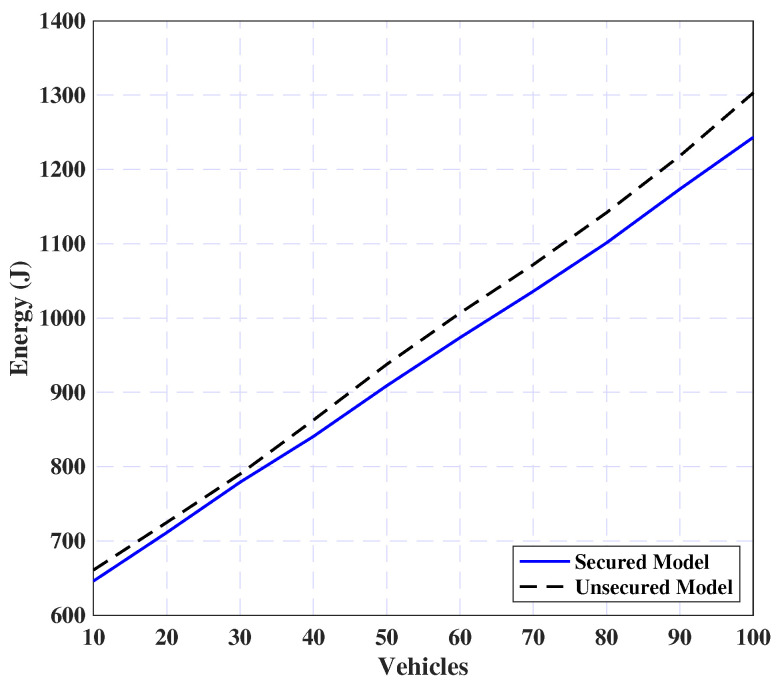
Performance impact of task security on the model.

This section evaluates the proposed model with and without adding the proposed security layer where the energy consumption is measured concerning the number of vehicles (see Figure 10). It is evident from the plot that the energy consumption increases almost linearly with the number of vehicles for both secured and unsecured models, while the secured model consistently incurs higher energy costs. This increase is primarily attributed to the computational overhead of applying the security mechanism, which includes encryption and decryption processes. Despite the increased energy overhead, integrating a security layer is essential for safeguarding sensitive information and mitigating potential security threats in-vehicle networks.

#### 5.2.5. System Performance

To assess the effectiveness of the proposed framework, a comparative analysis was undertaken through simulations conducted under other five scenarios, which are as follows:

1. Local Execution: In this approach, all computational tasks are executed exclusively on the vehicles without utilizing external resources for offloading.
2. RSU Execution: In contrast, this approach fully leverages the computational capabilities of nearby Roadside Units (RSUs), offloading all tasks for remote processing and relying entirely on their computing power.
3. Model in [55]: This approach adopts the task offloading model proposed by [55], which employs a decision-making mechanism to dynamically determine the close-optimal execution location for each task based on a pre-defined set of criteria and constraints.
4. Model in [56]: This approach builds upon the task offloading framework introduced in [56], which incorporates a decision-making mechanism to dynamically select the near-optimal execution location for each task by considering a predefined set of constraints and system parameters.
5. Model in [33]: This approach adopts the task offloading model proposed by [33], which employs a decision-making framework to dynamically select the most suitable execution site for each task based on predefined system constraints and operational parameters.

**Figure 11:**
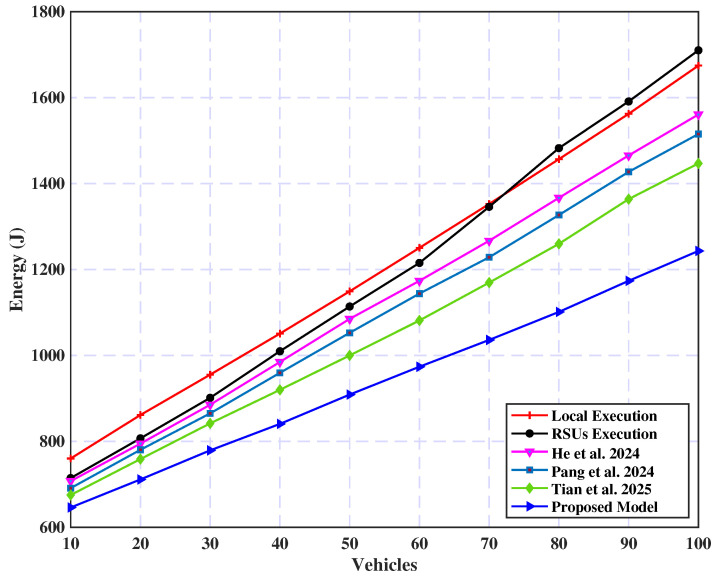
Energy consumption vs. vehicle count [33,55,56].

Figure 11 illustrates a comparative evaluation of energy consumption across different strategies as the number of vehicles increases. The data indicate a steady increase in energy consumption with higher vehicle counts. Notably, the proposed model demonstrates superior performance in energy efficiency compared with other strategies, particularly at larger vehicle scales. Although the RSU policy initially shows competitive energy consumption, it outperforms the local policy as vehicle numbers grow. Furthermore, the studies in [33,55,56] achieved improved performance as the number of vehicles increased; however, their effectiveness remains lower than that of our proposed model. This is due to increased demand for communication resources during offloading processes. Moreover, integrating load balancing and caching mechanisms in the proposed model is crucial in achieving lower energy consumption levels.

**Figure 12:**
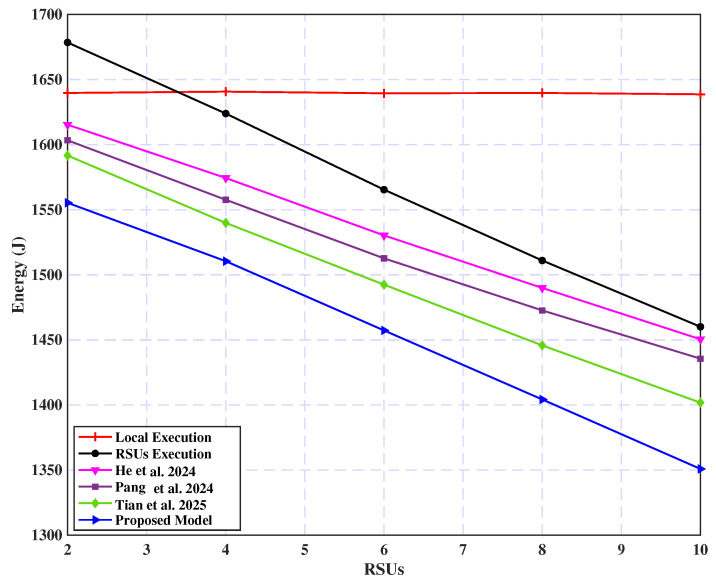
Energy consumption vs. RSU count [33,55,56].

Figure 12 analyzes the relationship between the number of RSUs and energy consumption across various execution strategies. The local execution strategy shows no change in energy consumption as the number of RSUs varies since it does not utilize RSU resources. Conversely, both the edge-based and proposed models exhibit a significant decrease in energy consumption with an increase in RSU count. The proposed model consistently achieves the highest energy efficiency, outperforming other strategies. This reduction in energy consumption is primarily due to the enhanced availability of computational resources provided by the increased number of RSUs. With more RSUs available, vehicles can offload tasks more effectively, thereby reducing the energy required for processing. The proposed model utilizes enhanced resource allocation to optimize task execution, leading to a more substantial reduction in energy consumption than other strategies.

**Figure 13:**
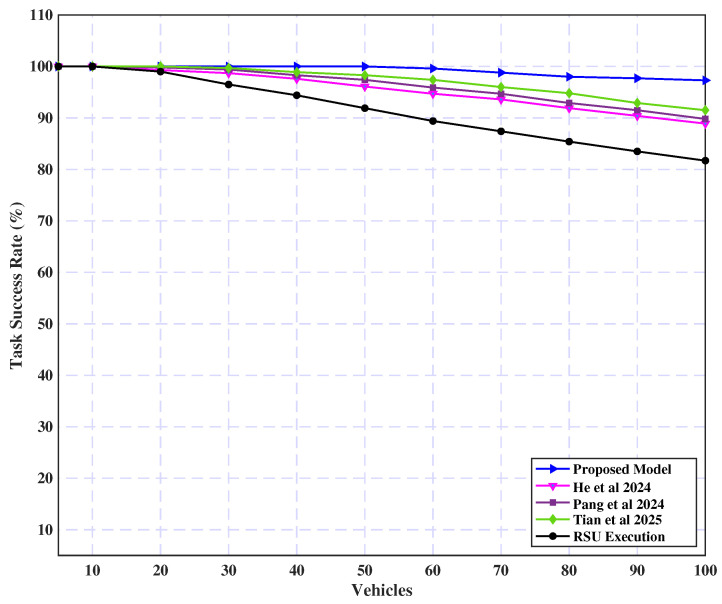
Task success rate (%) by vehicle count [33,55,56].

Finally, Figure 13 presents a comparative analysis of the task success rate (completed tasks as a percentage of total tasks) across varying vehicle counts. For fewer vehicles (fewer than 20), all three strategies achieve a 100% success rate. However, as the vehicle count increases, the success rate declines. Specifically, the success rate drops to 89%, 90%, 92%, and 82% for the strategies proposed in prior works [33,55,56] and RSU execution, respectively. In contrast, the proposed model maintains a higher success rate, reaching 97% even with 100 vehicles. This is attributed to the increased competition for available RSU resources in the other strategies as the number of vehicles grows. The proposed model, however, efficiently distributes the workload across servers and employs UAVs to enhance the utilization of edge server resources, thereby maintaining a higher task success rate under increased vehicular load.

While the proposed framework demonstrates significant improvements in energy efficiency, security, and load balancing, several challenges remain for real-world deployment. The dynamic nature of vehicular mobility introduces challenges in maintaining seamless connectivity and effective resource allocation. Network variability and latency fluctuations may impact task offloading performance, requiring adaptive mechanisms for real-time optimization. Additionally, while the AES-based security layer enhances data protection, challenges remain in securing key distribution and authentication mechanisms against sophisticated cyber threats. Furthermore, integrating UAVs for resource augmentation could face practical deployment challenges, including operational costs, airspace regulations, and energy limitations. Addressing these challenges in future work will ensure the framework’s feasibility in large-scale intelligent transportation systems.

## 6. Conclusions

This paper introduces a comprehensive, secure, and energy-efficient framework designed for VECC networks. The framework incorporates an advanced load-balancing algorithm that efficiently redistributes vehicles to balance the load across RSUs, thereby minimizing communication costs. Additionally, UAVs are employed as supplementary edge servers, providing communication and computation resources in densely populated areas where ground-based RSUs may be overwhelmed. A robust encryption layer utilizing AES cryptography combined with dynamic one-time key generation is implemented to ensure data security. Furthermore, a novel task-caching mechanism is introduced, which considers server capacity, task popularity, and data size to cache only pertinent application code and task data, thereby enhancing system efficiency by reducing redundant computations and optimizing resource utilization. The framework integrates computational offloading, load balancing, task caching, and security measures into an optimization problem to minimize vehicle energy consumption while satisfying the latency constraints. A reinforcement learning-based approach is employed to address this optimization challenge, enabling the determination of close-optimal solutions. Simulation results demonstrated the efficacy of the proposed framework, showcasing substantial reductions in energy consumption compared with existing benchmark models, thereby underscoring its effectiveness in optimizing resource management within VECC networks.

The proposed model will be expanded in future work to address bandwidth limitations, particularly for transmitting larger data sizes, by integrating a data compression layer. Additionally, we plan to enhance the model’s adaptability by incorporating dynamic traffic conditions, including variations in vehicle speed, sudden task peaks, and network fluctuations. This will involve integrating real-world vehicular mobility patterns and adaptive offloading strategies to evaluate system performance under highly dynamic environments. Furthermore, we will explore reinforcement learning-based approaches to optimize task scheduling and resource allocation in rapidly changing vehicular networks. An artificial intelligence-based system will also be developed to improve automated security decision making by incorporating user behavioral analysis. Finally, we will refine our blockchain integration by focusing on consensus mechanism design, ensuring efficient, decentralized security solutions that strengthen data integrity and privacy in vehicular networks.

## Figures and Tables

**Table 1:** Overview of VECC studies.

Ref.	Objective	Methodology	Security	Limitations
[24]	Optimize task completion time, energy consumption, resource costs, and load balance	Many-objective optimization algorithm for task offloading		Data security and privacy are not considered.
[25]	Optimize secure multi-user multi-task offloading in mobile edge computing for IoT.	Utilize integrated optimization of resource allocation, compression, and security for efficient MEC offloading.	✓	Lacks efficient load balancing among edge nodes. Task caching is not considered.
[26]	Minimize vehicular task delay under a long-term UAV energy constraint.	Utilize Lyapunov optimization and Markov approximation for real-time UAV-assisted vehicular task offloading optimization.		Resource distribution lacks task specificity. Data security and privacy are not considered.
[27]	Minimize energy consumption while ensuring secure task offloading.	Utilize AES encryption, fingerprint authentication, and load-balancing algorithms for secure and energy-efficient task offloading.	✓	Inefficient load balancing for non-intersection vehicles. Ignore task caching.
[28]	Minimize system cost.	An AGSP algorithm integrating PSO, GA, and SA for optimized UAV task offloading.		Lacks security considerations. Real-time adaptability. Scalability for large UAV networks.
[29]	Minimize system cost.	Utilize multi-agent DRL with actor-critic networks for distributed computation offloading in vehicular edge networks.		Training is limited by large state and variable time. Mobility is not considered. Data security and privacy are not considered.
[30]	Minimize the total system computational delay	An intelligent partial offloading scheme uses digital twins and clustering to optimize partial offloading.		Data security and privacy are not considered. Task caching is not considered.
[31]	Minimize total system delay.	A DDPG-based reinforcement learning algorithm for multi-user task offloading and resource allocation optimization.		Energy consumption is ignored. Data security and privacy are not considered.
[32]	Optimize secure task offloading while minimizing latency and energy consumption.	Utilize Proximal Policy Optimization (PPO)-based deep reinforcement learning for secure and efficient task offloading in MEC.	✓	Lacks efficient load balancing among edge nodes. Task caching is not considered.
[33]	Minimize the long-term computation overhead and energy consumption.	Employ Deep Reinforcement Learning for optimizing task caching and computation offloading in VECC.		Lacks efficient load balancing among edge nodes. Data security and privacy are not considered.
[34]	Minimize task service latency.	A double deep Q-network-based approach with dynamic offloading.		Data security and privacy are not considered.
[35]	Minimize long-term computation overhead	A multi-agent deep reinforcement learning framework with task migration for continuous, efficient vehicular service.		Lacks efficient load balancing among edge nodes. Data security and privacy are not considered. Task caching is not considered.
[36]	Optimize task offloading and caching using spatiotemporal prediction in vehicular networks.	Utilize spatiotemporal prediction, deep reinforcement learning, and digital twin technology for efficient task offloading and caching.		Lacks efficient load balancing among edge nodes. Data security and privacy are not considered. Task caching is not considered. Data security and privacy are not considered.
[37]	Minimize vehicular task delay.	A service-aware offloading strategy that utilizes real-world vehicular data for dynamic service prediction.		Lacks efficient load balancing among edge nodes. Data security and privacy are not considered. Task caching is not considered.
[38]	Optimize resource allocation and task offloading in high-mobility vehicular edge networks.	Utilize a Double Deep Q-Network (DDQN) with multi-agent collaboration for dynamic task offloading and resource allocation.		Lacks efficient load balancing, data security, and privacy measures. Task caching is not considered.
[39]	Optimize traffic rerouting and task offloading using federated learning and blockchain in IoV.	Utilize federated learning, blockchain, and hybrid ACO-DRL for secure task offloading and dynamic traffic rerouting.	✓	Load balancing and task caching among edge nodes are not considered.
[40]	Optimize task scheduling and offloading using federated learning and blockchain.	Utilize federated learning with blockchain for secure task scheduling and computational offloading in mobile cloud computing.	✓	Increased latency due to blockchain verification and consensus mechanisms. Scalability challenges in managing large user bases and diverse tasks. High energy consumption from frequent model updates and blockchain processing.
**Proposed**	Minimize energy consumption while considering a latency.	An energy optimization and security-aware deep reinforcement learning-enabled task offloading framework for multi-tier VECC networks.	✓	Vehicle mobility issues are not considered.

**Table 2:** Algorithm Calculations.

Vehicles	Input Size	CPU Cycles	Estimated Processing Time		Best RSU
			Transmission Time	Computation Time	
$V_{10}$	15	15	RSU→114.4	RSU→18.8	${\mathrm{RSU}}_{3}$
			RSU→34.8	RSU→32.0	
$V_{11}$	20	10	RSU→117.5	RSU→15.6	${\mathrm{RSU}}_{2}$
			RSU→24.8	RSU→21.5	
			RSU→38.0	RSU→32.5	
$V_{12}$	15	20	RSU→112.0	RSU→110.0	${\mathrm{RSU}}_{2}$
			RSU→24.8	RSU→24.0	
$V_{15}$	10	15	RSU→24.0	RSU→23.75	${\mathrm{RSU}}_{3}$
			RSU→33.2	RSU→33.0	
$V_{16}$	15	25	RSU→24.0	RSU→23.75	${\mathrm{RSU}}_{3}$
			RSU→33.2	RSU→33.0	

## Data Availability

The data is available upon request.
